# Characterization and Role of AP2/EREBP Genes with Decreasing Expression During Leaf Development in 84K Poplar

**DOI:** 10.3390/plants14182842

**Published:** 2025-09-11

**Authors:** Sanjiao Wang, Nan Liu, Jingna Si, Sihan Zhang, Xiaomin Liu

**Affiliations:** State Key Laboratory of Tree Genetics and Breeding, College of Biological Sciences and Technology, Beijing Forestry University, Beijing 100083, China; m17835729632@163.com (S.W.); nan474085373@bjfu.edu.cn (N.L.); jingnasi@bjfu.edu.cn (J.S.); baijiu051006@163.com (S.Z.)

**Keywords:** *AP2/EREBP* gene family, leaf development, 84K poplar, *Populus alba* × *Populus glandulosa*

## Abstract

The 84K poplar (*Populus alba* × *Populus glandulosa*) is a fast-growing hybrid poplar that was introduced from South Korea by the Chinese Academy of Forestry in 1984. To gain deeper insight into the regulatory mechanisms of leaf development in 84K poplar, we performed bulk RNA sequencing and found that numerous members of the AP2/EREBP family exhibited expression changes, suggesting their crucial roles in leaf development. The AP2/EREBP transcription factor family is one of the largest and most conserved gene families in plants. These genes play a crucial role in plant growth, development, and stress responses. In this study, we identified and analyzed 400 AP2/EREBP genes through transcriptome analysis, excluding genes with missing values (NAs) or FPKM < 1, and selected 76 genes based on their expression patterns at different stages of leaf development. The 76 genes were classified into three subfamilies based on phylogenetic analysis and structural domain characteristics: the RAV subfamily, the ERF subfamily, and the AP2 subfamily. Each subfamily shares similar gene structures and motifs while also exhibiting distinct differences. Segmental duplication events may have contributed to the evolution of this gene family. Most of the promoter cis-acting elements are related to light responses, with fewer elements associated with palisade tissues and hormones. Eight genes, selected for their gradually decreasing expression during leaf development, were validated through RT-PCR experiments. Among them, five genes—*Pop_G10G022861*, *Pop_A01G003858*, *Pop_A01G081120*, *Pop_A01G074798*, and *Pop_A07G010900*—exhibited a decreasing trend in expression across the three stages of leaf development. Subcellular localization analysis indicated that *Pop_A01G003858* and *Pop_G11G077730*, two randomly selected genes from the eight AP2/EREBP members validated by RT-PCR, are localized in the nucleus. In conclusion, these findings provide valuable insights into the evolutionary relationships of the 73 AP2/EREBP family members in 84K poplar leaves and lay a foundation for future studies on leaf development.

## 1. Introduction

During plant growth, external environmental factors, such as light, temperature, and water availability, frequently affect their development [1,2]. In response, plants have evolved a sophisticated system of transcriptional regulation, one component of which is a network controlled by transcription factors. Light is an important environmental signal, and it regulates leaf development in plants. Transcription factors, including HY5, PIF3, GBF1, and CPRF2, form a hierarchy of networks to mediate light signaling through the coordinated activation and repression of specific downstream genes [3]. Containing one or more specific DNA-binding domains, these factors play critical roles throughout the life cycle of higher plants [4,5]. In plants, 58 transcription factor families have been identified, including APETALA2/Ethylene Response Element Binding Factor (AP2/EREBP), bZIP, C2H2, MYB, MADS, NAC, and WRKY [4,6,7]. This paper focuses specifically on the AP2/EREBP superfamily.

The AP2/EREBP transcription factors are a class primarily found in plants but are also present in protists, cyanobacteria, and viruses [6,8,9]. This superfamily is one of the largest groups of transcription factors in the plant kingdom [10,11,12]. Members of the AP2/EREBP superfamily share a highly conserved AP2 DNA-binding domain, consisting of 60 amino acids, with the YRG element at the N-terminus and the RADY element at the C-terminus [4,8,10,13]. Based on the number of AP2 domains and sequence similarity, the AP2/EREBP genes are divided into three subfamilies: the AP2 subfamily, the related to ABI3/VP1 (RAV) subfamily, and the ethylene response factor (ERF) subfamily [2,14,15,16]. The AP2 subfamily contains two AP2 domains, the RAV subfamily consists of one AP2 domain and one B3 DNA-binding domain, and the ERF subfamily, the largest within the AP2/EREBP superfamily, is characterized by a single AP2 domain [16,17,18].

Numerous studies have demonstrated that the *AP2/EREBP* genes play a key regulatory role in various physiological processes, including plant morphogenesis, hormone signaling, and metabolite regulation [19,20,21,22]. The functions of these genes vary across different subfamilies. The AP2 subfamily is crucial for regulating flower development, establishing the floral meristem, and modulating seed development [9,18,23,24]. The RAV subfamily is involved in the plant’s response to various abiotic stresses [25,26], while the ERF subfamily participates in a wide range of processes in plants [16].

The functional characterization of the AP2/EREBP transcription factors has been extensively investigated in a variety of plants, including *Arabidopsis thaliana*, rice, wheat, soybean, tomato, poplar, and maize [1,4,12]. In *Arabidopsis*, PLT1 and PLT2 are key effectors in the establishment of the stem cell niche during embryonic pattern formation [27]. The *PUCHI* gene in the AP2 subfamily is required for a coordinated pattern of cell division during lateral root formation in *Arabidopsis thaliana* [28]. In rice, AP2/ERF-encoding genes regulate multiple molecular mechanisms of floral organ development, flowering time, grain size, and quality [7]. In wheat, transcription factors from the AP2 subfamily respond to heat stress, promoting better growth under stressful conditions [29]. In soybean, *GmDREB1* regulates the expression of downstream stress-related genes by forming a heterodimer with the ERF transcription factor, thereby enhancing drought tolerance in transgenic soybean [30]. During tomato fruit ripening, the expression levels of *SlERF4-10* and *SlERF7-3* were upregulated, suggesting a potential regulatory role during this stage. Moreover, the EAR motifs present in SlERF2-6, SlERF4-1, SlERF4-10, SlERF4-11, SlERF7-3, SlERF3-4, and SlERF10-1 proteins were found to repress the expression of their target genes by binding to DRE/CRT elements in the promoter regions [31]. *SlRAV2* in tomato regulates the expression of *SlERF5*, thereby enhancing the plant’s resistance to pathogen infection [32]. *PtoERF15*, induced by osmotic stress, and its target gene, *PtoMYC2b*, are involved in regulating blood vessel size, density, and cell wall thickness in response to drought in poplar [33]. In addition, *peSHN1*, *PagERF072*, *PagERF16*, and *PagCRF8* have been reported to play roles in drought tolerance, salt stress, and leaf development [34,35,36,37,38]. The results above indicate that AP2/EREBP transcription factors play essential roles in plant development and stress resistance.

During leaf development, chlorophyll biosynthesis, chloroplast division, and development are highly active, with certain regulators likely playing crucial roles in coordinating chloroplast activity and leaf development. Woody plants undergo leaf development annually, making them valuable systems for studying this process. The 84K poplar (*Populus alba* × *Populus glandulosa*) is particularly suitable for investigating leaf development in woody plants. It is a fast-growing hybrid species, known for its high-quality wood, absence of spring catkins, broad adaptability, and high genetic transformation efficiency. Owing to these characteristics, it has become an important model plant for studies on tree physiology and genetic engineering [39]. Here, we performed bulk RNA sequencing on 84K poplar leaves to identify key regulators involved in leaf development and found that AP2/EREBP transcription factors are closely associated with different developmental stages. We further applied bioinformatics analysis, semi-quantitative PCR, and transient transformation to investigate the roles and expression patterns of AP2/EREBP family members that exhibited a decreasing expression trend during leaf development.

## 2. Results

### 2.1. Transcriptomic Analysis and Gene Screening

**Figure 1:**
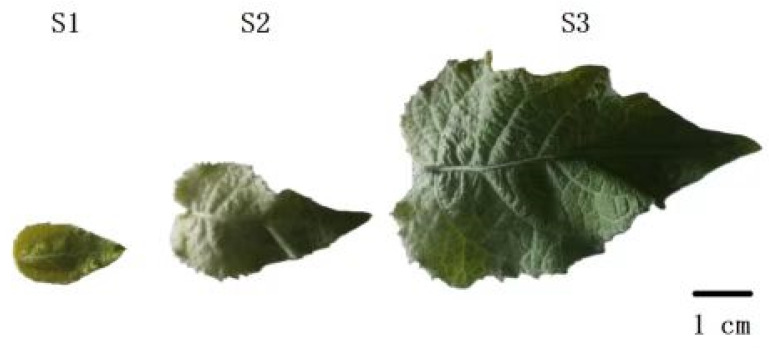
Morphology and stages of leaf development in 84K poplar. Based on the phenotypic characteristics of leaf development in 84K poplar, three key developmental stages were identified: the first stage, designated as S1, represents the early stage of leaf development, with a leaf length of approximately 2.4 cm; the second stage, designated as S2, corresponds to developmental phase with rapid leaf growth, reaching a length of around 4.7 cm; and the third stage, designated as S3, marks the maturation of the leaf, with a leaf length reaching approximately 8.3 cm.

Leaf development in 84K poplar was classified into three distinct stages: the early stage (S1), intermediate stage (S2), and mature stage (S3) (Figure 1). Samples from these three stages were used for transcriptome sequencing. Our bulk RNA sequencing data revealed that more than 400 genes in the AP2/EREBP family are associated with leaf development in 84K poplar. After excluding genes with missing values (NAs), 331 genes with valid FPKM data were retained. We then applied a threshold by removing genes with FPKM < 1, leaving 233 genes. The remaining genes were differentially expressed at various stages of leaf development and were screened based on their expression patterns across three stages (with a decreasing trend in expression and FPKM mean values ≥ 1), resulting in 76 genes (Table 1). Subsequentlgy, we conducted a detailed analysis of these 76 genes.

### 2.2. Phylogenetic Analysis of AP2/EREBP Proteins in 84K Poplar

**Figure 2:**
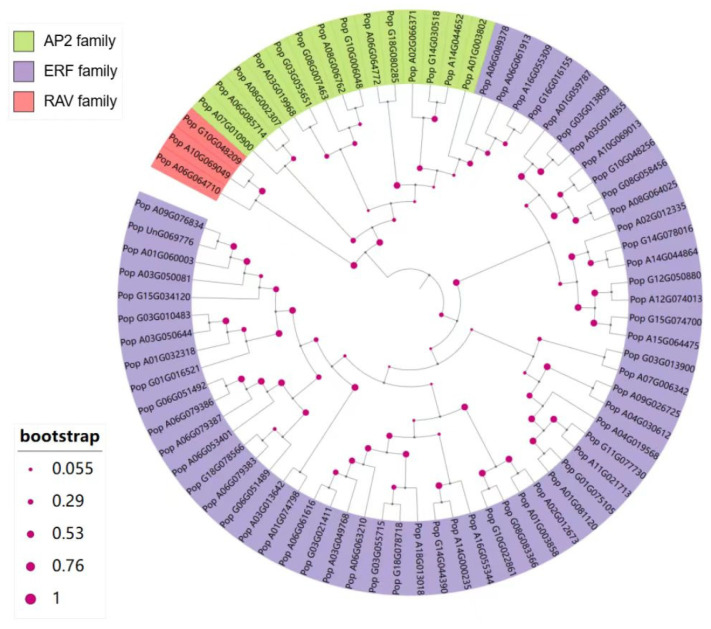
Phylogenetic analysis of AP2/EREBP proteins in 84K poplar. Different background colors represent three groups: red indicates the RAV subfamily, green indicates the AP2 subfamily, and purple indicates the ERF family. Bootstrap values are shown as magenta circles on branches, with 1000 bootstrap replications performed.

To investigate the evolutionary relationships of the 76 AP2/EREBP proteins in 84K poplar, we constructed a phylogenetic tree using the neighbor-joining method and the JTT model in MEGA11 [40]. As shown in Figure 2, the 76 AP2/EREBP proteins were divided into three different subfamilies: the RAV subfamily, the ERF subfamily (including both ERF and DREB members), and the AP2 subfamily. Among these, the RAV subfamily contains the fewest genes, with only three, while the ERF subfamily includes the most genes, totaling 59. The AP2 subfamily consists of 14 genes. These results indicate that the AP2/EREBP superfamily in 84K poplar shares the same characteristic observed in other species, namely, that the ERF subfamily is the largest and contains the most members. Additionally, these results suggest that the functions of these genes may differ between subfamilies. In the phylogenetic tree, the RAV subfamily formed a sister group to the AP2 subfamily clade, while the AP2 and ERF subfamilies did not fully separate. Some members of the ERF subfamily are closely related to the AP2 subfamily.

### 2.3. Conserved Domain and Motif and Gene Structure Analysis of AP2/EREBP in Poplar

**Figure 3:**
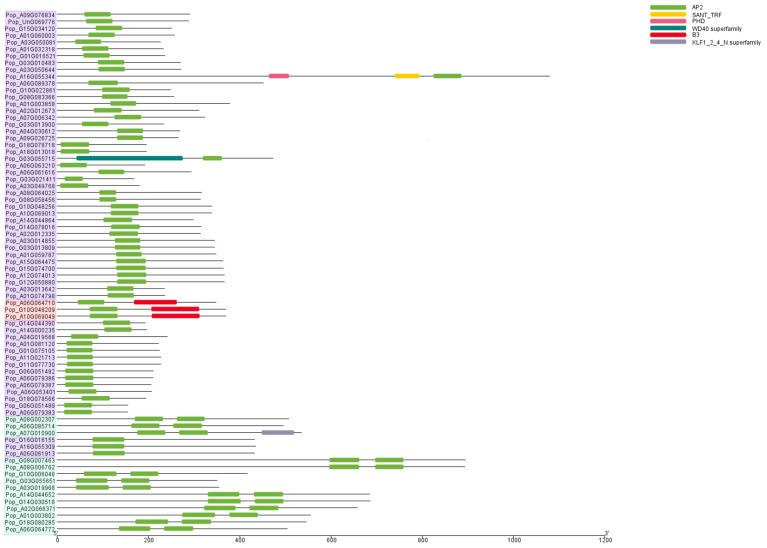
Analysis of conserved domains within AP2/EREBP proteins. Different colors represent distinct domains. Different background colors represent three groups: red indicates the RAV subfamily, green indicates the AP2 subfamily, and purple indicates the ERF family.

Conserved domain analysis is essential for understanding the evolutionary relationships and functions of distinct gene subfamilies. To investigate the similarities and differences in the AP2/EREBP proteins, we performed conserved domain analysis using the NCBI CDD. We identified six conserved structural domains within the AP2/EREBP proteins: AP2; KLF1 (krueppel-like factor 1); B3 (basic region 3); SANT (switching-defective protein 3 (Swi3), adaptor 2 (Ada2), nuclear receptor co-repressor (N-CoR), transcription factor (TF)IIIB); PHD (plant homeodomain); and WD40 (40–60 amino acids having tryptophan (W)–aspartic acid (D)) (Figure 3). All proteins contain the AP2 domain. In addition to the AP2 domain, Pop_A10G069049, Pop_A06G064710, and Pop_G10G048209 also contain the B3 domain, which groups them into the same subfamily: the RAV subfamily. Pop_A08G002307, Pop_A06G085714, and 12 other proteins possess two AP2 domains, thereby grouping them into the AP2 subfamily. The remaining proteins each contain a single AP2 domain, forming the largest group: the ERF subfamily. Moreover, besides the AP2 domain, Pop_A16G055344 contains both a SANT domain and a PHD domain, while Pop_G06G055715 contains the WD40 domain, suggesting functional differences from other members in the same subfamily.

**Figure 4:**
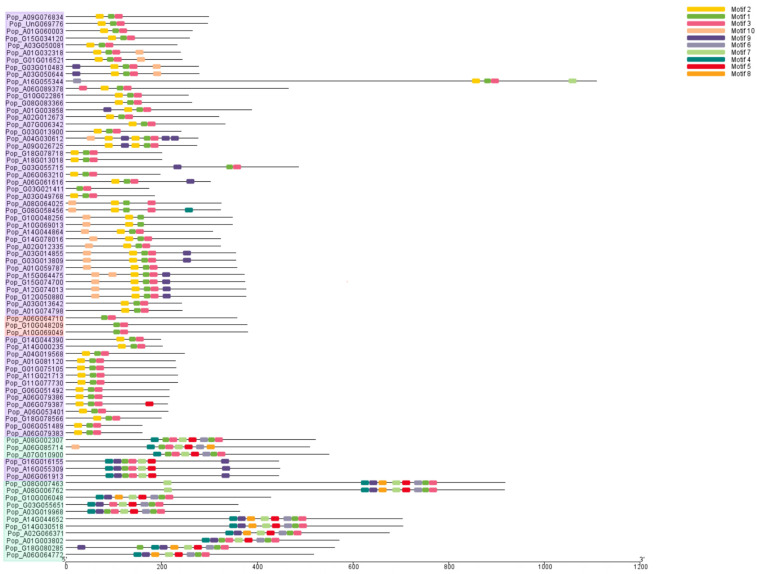
Analysis of the conserved motifs in the 76 AP2/EREBP proteins. Different motifs are represented by differently colored rectangles. Different background colors represent three groups: red indicates the RAV subfamily, green indicates the AP2 subfamily, and purple indicates the ERF family.

Next, we analyzed the conserved motifs of the 76 AP2/EREBP proteins in poplar using the MEME online server. As shown in Figure 4, 10 conserved motifs were identified and labeled as Motifs 1 to 10. All 76 proteins contain conserved motifs, but proteins in different subfamilies possess distinct motifs. Pop_A10G069049, Pop_A06G064710, and Pop_G10G048209 members in the RAV subfamily contain two motifs: Motif 1 and Motif 3. Pop_A08G002307, Pop_A06G085714, and 12 other proteins in the AP2 family possess Motifs 1, 3, 4, 5, 6, and 7. However, there are minor differences among proteins in the AP2 subfamily. For example, Pop_A08G002307, Pop_A06G085714, and Pop_A07G010900 lack Motif 9, while the other 11 proteins in this subfamily contain Motif 9. In the ERF subfamily, all members contain Motif 1 and Motif 3. Additionally, with the exception of Pop_G16G016155, Pop_A16G055309, and Pop_A06G061913, the remaining proteins possess Motif 2. Pop_G16G016155, Pop_A16G055309, Pop_A06G061913, and Pop_G08G058456 contain the specific Motif 4.

**Figure 5:**
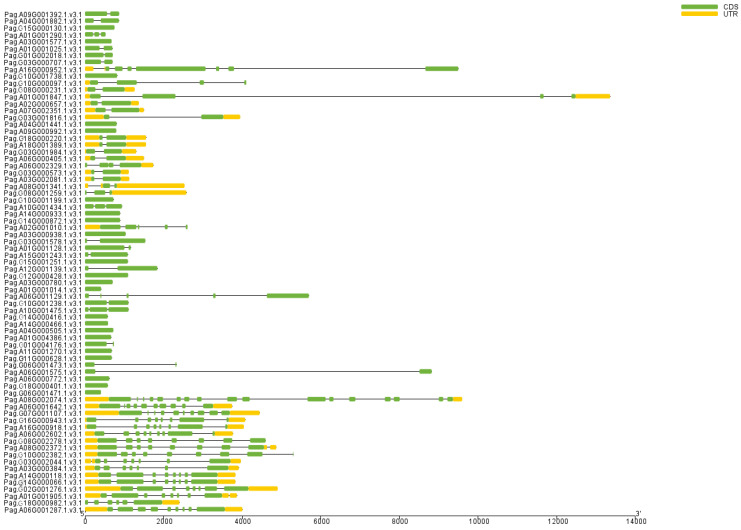
Analysis of *AP2/EREBP* gene structures. Yellow rectangles represent UTRs (untranslated regions), green rectangles represent CDS (coding sequences), and black lines represent introns.

We further analyzed the gene structures of the corresponding *AP2/EREBP* genes in 84K poplar. First, we aligned the 76 AP2/EREBP proteins with the protein sequences translated from the 84K poplar genome sequencing data and identified the corresponding genes using TBtools software with default parameters (Table 1) [41,42]. The 76 AP2/EREBP transcripts corresponded to 73 genes in 84K poplar genome. We then analyzed the exon–intron structures of these identified genes. The results showed that the AP2 subfamily contained more exons and introns compared to the other two subfamilies, with at least eight exons and seven introns (Figure 5). The three genes in the RAV subfamily contain variable numbers of exons, ranging from two to five, and introns, varying from one to four. Genes in the ERF subfamily exhibited a range of one to nine exons and zero to eight introns. Among the 59 genes, *Pag.A06G002602*, *Pag.G16G000943,* and *Pag.A16G000918* contain more exons and introns, with eight or more exons and seven or more introns, while the remaining genes contain fewer than seven exons and six or fewer introns.

### 2.4. Distribution of AP2/EREBP Genes on Chromosomes in 84K Poplar

**Figure 6:**
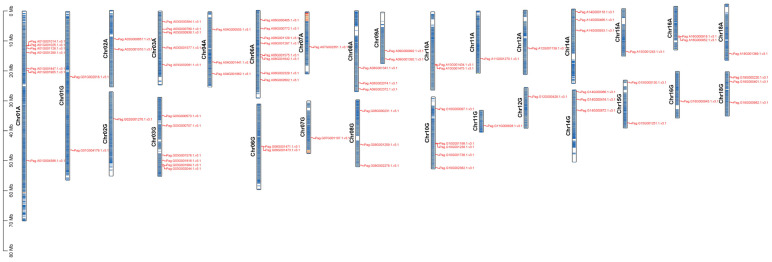
Chromosomal localization of the *AP2/EREBP* genes in 84K poplar. 84K poplar has 38 chromosomes, with scale bars in Mb, and chromosome numbers are indicated above the corresponding chromosomes. Different chromosomal colors represent varying gene densities, with red indicating the highest density and blue the lowest.

We investigated the distribution of the 73 AP2/EREBP genes across chromosomes in 84K poplar. Chromosomal localization analysis revealed that 76 AP2/EREBP genes were unevenly distributed across 28 chromosomes (Figure 6). No AP2/EREBP genes were detected on chromosomes Chr04G, Chr05A, Chr05G, Chr09G, Chr13A, Chr13G, Chr17A, Chr17G, Chr19A, and Chr19G. The number of genes distributed across chromosomes varied. Chromosome Chr06A contained the most genes, with eight genes, followed by chromosomes Chr01A and Chr03G, which carried seven and six genes, respectively. In contrast, chromosomes Chr02G, Chr07A, Chr07G, Chr11A, Chr11G, Chr12A, Chr12G, Chr15A, Chr16G, and Chr18A each contained only one gene.

### 2.5. Analysis of Cis-Acting Elements in AP2/EREBP Promoter Regions

**Figure 7:**
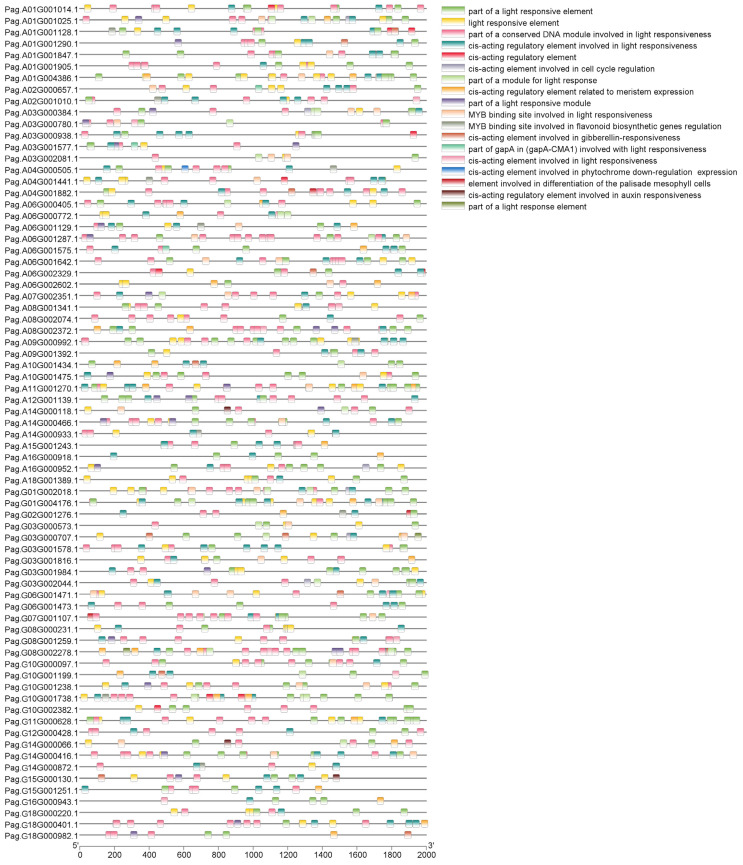
Cis-acting elements in promoter regions of *AP2/EREBP* genes (2000 bp upstream of the start codon). Differently colored squares represent distinct cis-acting regulatory elements.

To determine the distribution of cis-acting elements in the promoters of the 73 *AP2/EREBP* genes, we extracted 2000 bp upstream of the ATG from the promoters of 76 *AP2/EREBP* genes and analyzed the cis-acting regulatory elements in each. The results showed that the 76 *AP2/EREBP* genes contained 11 light-response-related elements, along with a small number of elements related to the differentiation of the palisade mesophyll cells and growth hormones (Figure 7). Light plays a crucial role in photosynthesis, as well as in the growth and development of leaves, while palisade tissues are essential components of leaf structure. All of these elements are important for leaf growth and development.

### 2.6. Collinearity and Duplication Analysis of AP2/EREBP Genes

**Figure 8:**
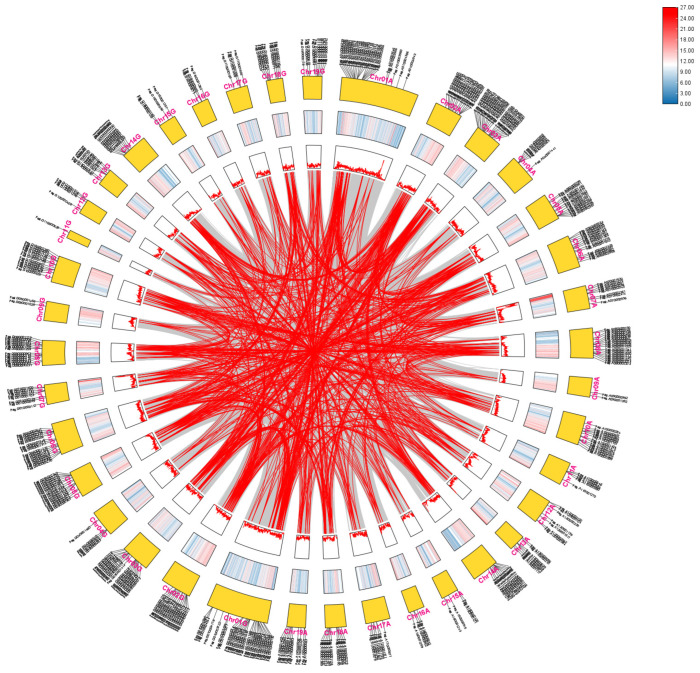
A circular presentation of the genomic map of 84K poplar. The outer segments of the circle represent the subgenomes A (right) and G (left), with each segment labeled from chromosome 1 (Chr01) to chromosome 19 (Chr19). Moving inward from the outermost part of each chromosomal segment, the first circle represents the gene positions in chromosomes. Adjacent to this, the gene density on each chromosome is visualized, with peaks indicating regions of higher gene concentration. The gray lines in the innermost circle represent all gene pair replications in the 84K genome, while the red lines highlight the *AP2/EREBP* collinear gene pairs.

To examine potential gene duplication and amplification events of the *AP2/EREBP* genes, we performed a collinearity analysis. The AP2/EREBP proteins from *Arabidopsis thaliana* were aligned with protein sequences translated from the 84K genome, identifying 398 AP2/EREBP members in 84K poplar. These 398 genes were subsequently used for intraspecific collinearity analysis. The results indicated extensive collinearity within the poplar *AP2/EREBP* gene superfamily (Figure 8). Most of the collinearly related genes were located on different chromosomes. *AP2/EREBP* genes on Chr03A were collinear with genes on Chr01A, Chr02G, Chr06A, Chr12A, and Chr18G, while some *AP2/EREBP* genes on Chr14A showed strong collinearity with genes on Chr02A. Interestingly, chromosomes such as Chr09G and Chr11G contained the fewest AP2/EREBP genes and exhibited fewer gene duplication and amplification events, whereas chromosomes including Chr01A, Chr02A, Chr03A, Chr01G, Chr02G, Chr03G, and Chr06G harbored more AP2/EREBP genes and showed extensive duplication and amplification events. Furthermore, segmental duplications in 84K poplar are the primary mechanisms responsible for amplification of *AP2/EREBP* genes.

**Figure 9:**
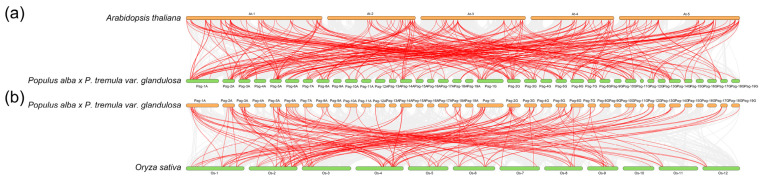
Synteny analysis of *AP2/EREBP* genes between 84K poplar and two representative plants. (**a**) Collinearity analysis between 84K poplar and *Arabidopsis thaliana*; (**b**) Collinearity analysis between 84K poplar and *Oryza sativa*. Gray lines indicate regions of collinearity for all gene pairs between the 84K poplar genome and those of other plants, while red lines highlight the collinear AP2/EREBP gene pairs.

To assess the collinearity of *AP2/EREBP* genes across species, we performed a collinearity analysis between these genes in 84K poplar and those in various plant species. The dicot *Arabidopsis thaliana* and the monocot *Oryza sativa* were selected for further analysis. The results revealed that multiple homologous gene pairs were present between 84K poplar and *Arabidopsis thaliana*, while fewer homologous gene pairs were found between 84K poplar and *Oryza sativa* (Figure 9). This suggests that 84K poplar and *Arabidopsis thaliana* share a greater number of collinear gene pairs compared to monocot species. These may be explained by the closer phylogenetic relationships between 84K poplar and *Arabidopsis thaliana* relative to *Oryza sativa*. Moreover, whole-genome duplication events may have contributed to the expansion of the AP2/EREBP gene family from *Oryza sativa* to 84K poplar and *Arabidopsis thaliana*.

### 2.7. Analysis of the Expression Levels of 76 AP2/EREBP Genes

**Figure 10:**
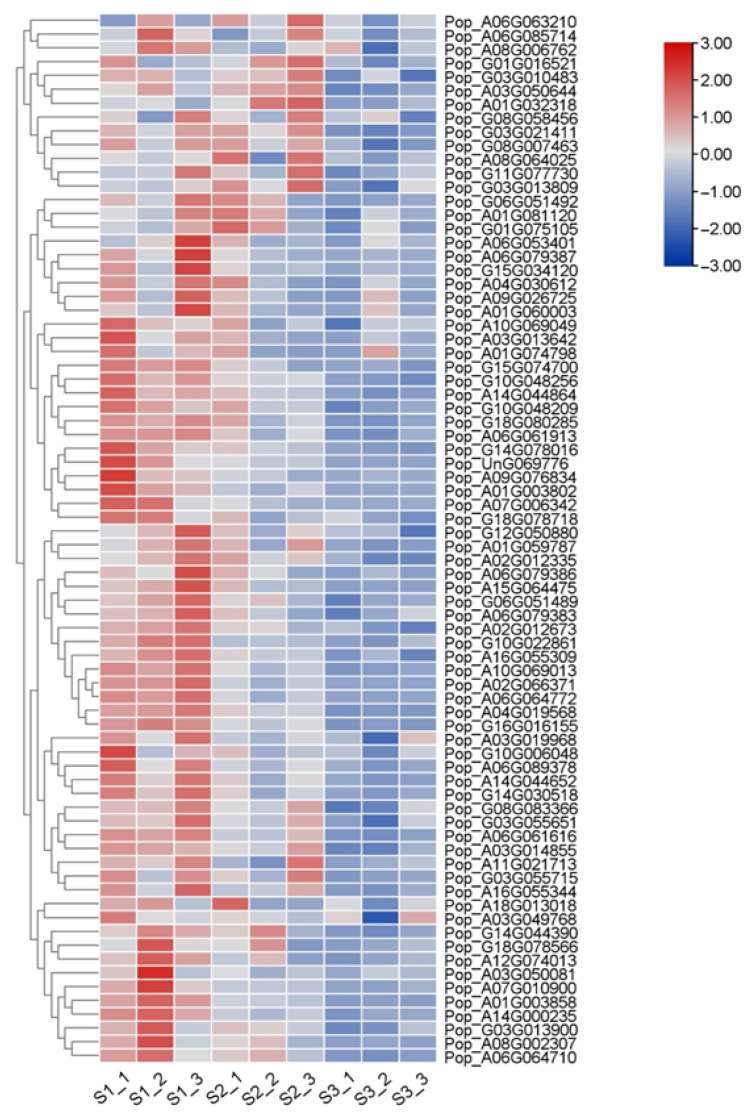
Expression profiles of 76 *AP2/EREBP* genes at different leaf developmental stages. The schematic diagram illustrates the expression levels, with red representing high expression and blue indicating low expression. Each developmental stage included three biological replicates.

We further analyzed the gene expression levels of AP2/EREBP genes in 84K poplar. The expression levels of 76 AP2/EREBP genes were derived from our transcriptome sequencing data of 84K poplar leaves at three different developmental stages. These expression levels are displayed in a heatmap (Figure 10). The results showed that the expression levels of AP2/EREBP genes varied across different growth stages of the leaves, with an overall decreasing trend. This was particularly evident in eight genes: Pop_G11G077730, Pop_A01G081120, Pop_G10G022861, Pop_A08G002307, Pop_A03G013642, Pop_A01G074798, Pop_A01G003858, and Pop_A07G010900.

### 2.8. Validation of Expression Levels for Eight AP2/EREBP Genes in 84K Poplar

**Figure 11:**
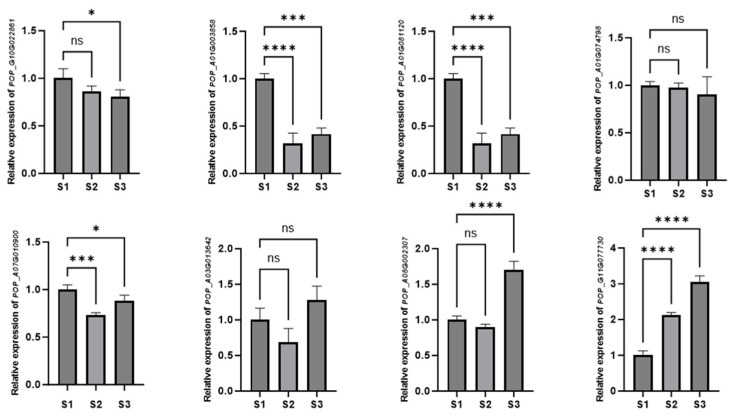
qPCR analysis of the expression levels of eight *AP2/EREBP* genes at three different developmental stages (S1, S2, and S3) of leaves. *PagPP2AA3* was used as the internal control, and three biological replicates were performed. Symbols indicate significance levels: * *p* ≤ 0.05; *** *p* ≤ 0.001; **** *p* ≤ 0.0001; ns indicates no significant difference (*p* > 0.05).

To validate the bulk RNA sequencing data, we selected eight genes with significantly altered expression levels across three leaf developmental stages and analyzed their transcriptional levels using real-time quantitative PCR (qPCR). As shown in Figure 11, the expression patterns of these eight genes exhibited three distinct trends. Among them, five genes—*Pop_G10G022861*, *Pop_A01G003858*, *Pop_A01G081120*, *Pop_A01G074798*, and *Pop_A07G010900*—displayed a decreasing expression trend across the three developmental stages, although the expression level of *Pop_A01G074798* showed no obvious change. The expression levels of *Pop_A03G013642* and *Pop_A08G002307* followed a fluctuating trend, initially decreasing and then increasing. In contrast, *Pop_G11G077730* showed a consistently increasing trend in expression.

**Figure 12:**
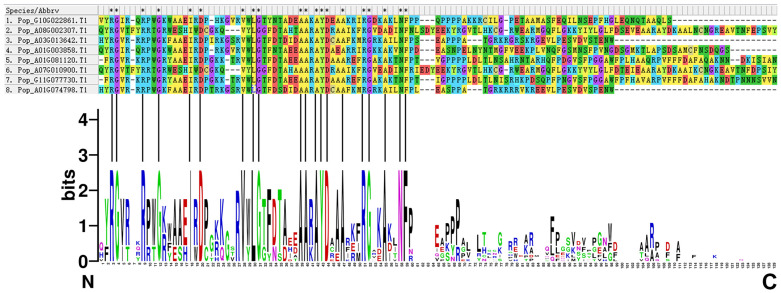
Sequence alignment and logo of the eight AP2/EREBP proteins. The upper panel displays the multiple sequence alignment of the eight proteins, while the lower panel shows sequence logo for the AP2 domains of the eight AP2/EREBP proteins. Asterisks (*) indicate positions that are 100% conserved in the alignment.

The characteristics of the eight AP2/EREBP proteins were analyzed. Multiple sequence alignment of the full-length sequences revealed that all eight proteins contain complete and highly conserved AP2 structural domains (Figure 12).

### 2.9. Subcellular Localization of Two AP2/EREBP Proteins in Tobacco

**Figure 13:**
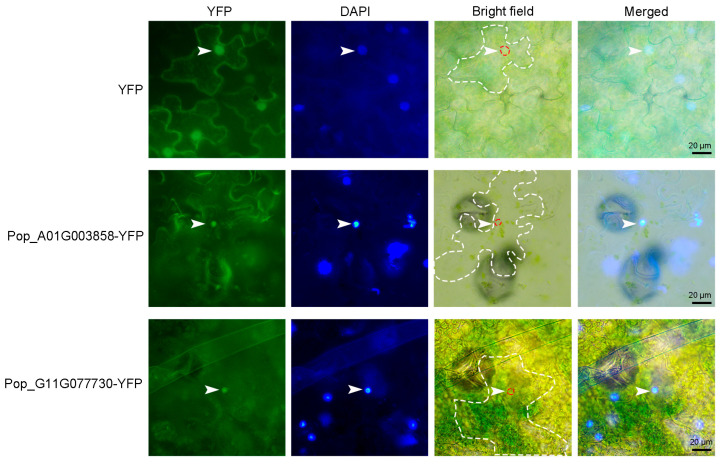
Analysis of the subcellular localization of Pop_A01G003858 and Pop_G11G077730. *35S: YFP*, *35S: Pop_A01G003858-YFP,* and *35S: Pop_G11G077730-YFP* were infiltrated into tobacco leaves, and fluorescence signals were observed 48 h post-infiltration. The *35S: YFP* construct was used as a control. DAPI, a DNA-binding dye used for nuclear staining, was applied at a working concentration of 5 μg/mL for 15–20 min. White arrowheads indicate nuclei. The white dotted line outlines epidermal cells, and the red dotted line marks nuclei in the bright-field images. Scale bar represents 20 μm.

To investigate the subcellular localization of *AP2/EREBP* genes, two genes—*Pop_A01G003858* and *Pop_G11G077730*—were randomly selected for analysis. The cDNA sequences of *Pop_A01G003858* and *Pop_G11G077730* were each fused to the N-terminus of YFP under the control of the CaMV 35S promoter. These constructs were then introduced into tobacco leaves via *Agrobacterium*-mediated transient transformation to analyze the subcellular localization of the two proteins. As shown in Figure 13, YFP fluorescence signals from Pop_A01G003858 and Pop_G11G077730 were specifically observed in the nucleus, co-localizing with the nuclear stain DAPI, whereas the control YFP signal was distributed throughout both the cytoplasm and the nucleus. These results indicate that *AP2/EREBP* genes encode nuclear-localized transcription factors, suggesting that they play regulatory roles within the nucleus.

## 3. Discussion

Previous studies have reported the presence of AP2/EREBP family transcription factors in species such as *Arabidopsis thaliana* [43], poplar [44], cucumber [45], and wheat [46], where they function in stress responses, including drought, low temperature, and heat stress. In this study, bulk RNA sequencing of 84K hybrid poplar identified more than 400 *AP2/EREBP* transcripts related to leaf development. In *Arabidopsis thaliana*, 147 AP2/ERF members have been identified [47], while 170 *AP2/ERF* transcription factors have been reported in rice [48] and 200 *AP2/ERF* genes in *Populus trichocarpa* [49]. By contrast, we identified over 400 *AP2/EREBP* transcripts in 84K poplar, which may be attributed to its hybrid nature, as allelic variation could nearly double the number of transcripts compared with the *AP2/ERF* genes in *Populus trichocarpa*. In addition, alternative splicing of some genes may also contribute to the elevated number of *AP2/EREBP* transcripts in 84K poplar.

Among the ~400 *AP2/EREBP* transcripts associated with poplar leaf development, 76 exhibited a decreasing expression trend. Based on phylogenetic analysis and subsequent domain analysis, we categorized 76 AP2/EREBP proteins into three groups: AP2 subfamily, ERF subfamily, and RAV subfamily, with 14, 59, and 3 members, respectively. The *AP2/EREBP* genes exhibit similar classifications in other species. In wheat, the gene subfamilies DREB, ERF, AP2, RAV, and soloist consist of 57, 47, 9, 3, and 1 gene(s), respectively [50]. A total of 148 AP2/EREBP proteins have been reported in soybeans, including 26 members of the AP2 subfamily, 98 members of the ERF (ERF and DREB) subfamily, and 2 members of the RAV subfamily, excluding members of the soloist subfamily [51]. This study analyzed the conserved domain structure of AP2/EREBP proteins. According to previous studies, members of the AP2 subfamily contain two repeated AP2 domains, while proteins of the ERF and DREB subfamilies possess a single conserved AP2 domain [16,17,52]. These proteins can also be further divided into two subgroups: ERF and CBF/DREB. Members of the ERF subgroup are responsible for binding to the core motif, while members of the CBF/DREB subgroup recognize cis-acting elements [33,45,53]. In this study, a detailed classification reveals that *Pop_G16G016155*, *Pop_A16G055309,* and *Pop_A06G061913* belong to the CBF/DREB subgroup and may combine with cis-acting elements to regulate leaf growth. In addition, members of the RAV subfamily contain both a B3 domain and an AP2 DNA-binding domain, enabling them to specifically bind to the promoters of target genes with consensus motifs and regulate plant growth and leaf senescence [4,54].

In different plant species, certain motifs may be highly conserved. In this study, however, significant differences were observed in the motifs of different subfamilies in the phylogenetic tree. Proteins within the same subfamily share similar motifs, indicating conserved structures and functions, which may be genetically similar and share a common ancestor [55]. Introns play an important role in regulating gene transcription and contribute to gene expression. Therefore, studying gene structure can provide valuable insights into their functions. The results of this study show that most genes contain both introns and exons. Notably, the AP2 subfamily contains a higher number of introns and exons compared to other subfamilies. The introns and exons of different subfamilies vary in position and length, indicating functional differences [2,56]. In this study, 76 genes were distributed across 28 chromosomes, with varying numbers of genes located on each chromosome. Chr06A contains the highest number of genes, while Chr02G, Chr07A, Chr07G, Chr11A, Chr11G, Chr12A, Chr12G, Chr15A, and Chr16G each contain only one gene. Interestingly, some longer chromosomes have fewer genes, while short chromosomes harbor more genes, which may also be linked to their respective functions.

By predicting the cis-acting elements of the 76 genes, we can gain a deeper understanding of gene composition and facilitate the study of their functions [57,58]. In this study, we identified a variety of cis-acting elements, with light-response-related elements being the most abundant, followed by elements related to the differentiation of the palisade mesophyll cells and auxin response. Light [59], the differentiation of palisade mesophyll cells [60], and auxin [61] are all directly associated with the growth and development of plant leaves. Hence, we speculate that the *AP2/EREBP* gene family may be directly implicated in the growth and development of leaves. Existing studies on *Arabidopsis thaliana* have shown that AP2/EREBP is linked to rapid retrograde signaling in response to high light, further supporting our hypothesis [62]. To investigate gene duplication and amplification events of *AP2/EREBP*, we carried out collinearity analysis. Extensive collinearity was observed within the *AP2/EREBP* genes, suggesting that segmental duplication and whole genome duplication may have been the primary driver behind the expansion of the gene family in 84K poplar. Notably, the collinearity of *AP2/EREBP* genes between 84K poplar and *Arabidopsis thaliana* is significantly higher than that between 84K poplar and *Oryza sativa*. Several chromosomes of 84K poplar, including 4A, 9A, 13A, 15A, 16A, 9G, 11G, 13G, 15G, and 16G, lack collinear *AP2/EREBP* genes with *Oryza sativa*.

We analyzed transcriptome data from three different developmental stages of 84k leaves and identified 76 genes with a decreasing expression trend. Eight of these genes were selected for qPCR analysis and validation. It is worth noting that the expression levels of these genes varied at different stages of leaf development, and the expression patterns did not align with transcriptome data. Among them, *Pop_A08G002307* and *Pop_A07G010900* belong to the AP2 subfamily and exhibit fluctuating and decreasing expression trends across the three developmental stages, respectively, suggesting that they may play important regulatory roles during late and early stages of leaf development. *Pop_A01G081120*, *Pop_G10G022861*, *Pop_A01G0003858*, *Pop_A01G074798*, *Pop_G11G077730*, and *Pop_A03G013642* belong to the ERF subfamily, with their expression levels showing variability across the stages, indicating their complex roles in leaf development.

Transcription factors are typically localized in the nucleus, where they perform their regulatory functions. Previous studies have reported that AP2/ERF family members, such as ORA59 and RAP2.3, function in the nucleus [63]. In this study, we randomly selected two genes, *Pop_A01G003858* and *Pop_G11G077730*, to examine their subcellular localization. The results confirmed that these proteins are specifically localized in the nucleus, suggesting that they may play important regulatory roles during leaf development.

## 4. Materials and Methods

### 4.1. Plant Materials and Transcriptome Analysis

The hybrid poplar (*Populus alba* × *Populus glandulosa* clone 84K) was used in this study. Stems of 84K poplar, grown for one month, were propagated from microcuttings in bottles and cultured on rooting medium containing half-strength MS (1/2 MS) (Caisson Laboratories, Smithfield, UT, USA), 30 g L^−1^ sucrose, 0.02 mg L^−1^ naphthylacetic acid (NAA) (Biorigin (Beijing) Inc., Beijing, China), 5.5 g L^−1^ agar, and 0.05 mg L^−1^ indolebutyric acid (IBA) (Biorigin (Beijing) Inc., Beijing, China)in a greenhouse at 25 ± 1 °C with 16 h/8 h light/dark photoperiod (60 ± 5 μmol photons m^−2^ s^−1^) and 55 ± 5% relative humidity.

The poplar plantlets, grown on rooting medium until they reached a height of 8 cm, were then transferred to soil and allowed to continue growing for two additional months in the same greenhouse. Leaves from these plants, after two months of growth, were used as experimental materials. We selected plants in good growth condition and extracted total RNA at three leaf developmental stages: the early stage (S1), intermediate stage (S2), and mature stage (S3), followed by transcriptome analysis. Each experiment was performed in triplicate.

Sequencing was performed using the Illumina Hiseq2500 (Solexa) platform (Illumina, San Diego, CA, USA). A de novo transcriptome assembly was conducted, and gene and transcript expression levels were calculated using RSEM and represented by FPKM values. These data enabled a comprehensive analysis of the quality of the transcriptome sequencing.

### 4.2. Evolutionary Tree Analysis of AP2/EREBP Proteins

The 76 AP2/EREBP protein sequences were aligned and analyzed using MEGA software (version 11) [40]. The phylogenetic tree was constructed using the neighbor-joining method and the Jones–Taylor–Thornton (JTT) model. The tree was then refined using the online tool iTOL (https://itol.embl.de/, accessed on 21 December 2024) [64].

### 4.3. Prediction of AP2/EREBP Conserved Motifs

Conserved motifs of AP2/EREBP proteins were predicted using MEME web server (https://meme-suite.org/meme/tools/meme, accessed on 16 August 2024) with the motif parameter set to 10 [65], and the MAST 593 XML output file was downloaded and visualized using TBtools-II (v2.332) [66].

### 4.4. AP2/EREBP Gene Structure Analysis

The 76 AP2/EREBP protein sequences were aligned to the 84K poplar genome to identify their corresponding gene IDs and sequences. The gene sequences were then extracted and analyzed for exon–intron structure using the TBtools-II tool [66].

### 4.5. AP2/EREBP Structural Domain Analysis

The structural domains within the AP2/EREBP amino acid sequence were analyzed using the Batch-CDsearch function of the NCBI website (https://www.ncbi.nlm.nih.gov/Structure/bwrpsb/bwrpsb.cgi, accessed on 25 August 2024) [67].

### 4.6. Chromosomal Localization of AP2/EREBP

Chromosomal localization of the 84K *AP2/EREBP* genes was determined by analyzing the whole genome and annotation files using TBtools-II software [66].

### 4.7. Gene Duplication and Syntenic Analysis

Tandem duplication events among the ‘84K’ AP2/EREBP genes were identified by utilizing TBtools-II and MCScanX tools [66]. Segmental duplication events and interspecies gene synteny were then analyzed using TBtools-II, MCScanX, and BLASTP (version 2.15.0) [66].

### 4.8. Vector Construction and Transient Expression in Tobacco

Two constructs, *35S: Pop_A01G003858-YFP* and *35S: Pop_G11G077730-YFP*, were generated and transformed into *Agrobacterium tumefaciens* (Supplementary Table S1). After 48 h of incubation at 28 °C, single colonies were selected for further culture by shaking in liquid medium. After shaking for approximately 12 h, the prepared IM and AB salt solutions were mixed at a 19:1 ratio. Then, 200 μL of *Agrobacterium* culture was added to 2 mL of the IM/AB mixture and incubated at 28 °C with shaking at 200 rpm for an additional 4–8 h. The bacterial suspension was collected before the OD_600_ reached 0.8 and diluted with the IM/AB mixture to an OD_600_ of 0.2. The suspension was then slowly infiltrated into the abaxial side of tobacco leaves using a 1 mL sterile syringe. After infiltration, the plants were incubated in the dark for 48 h. Leaf segments from the infiltrated areas were then excised, mounted on slides, and observed under a fluorescence microscope.

### 4.9. RNA Extraction and Real-Time Quantitative PCR (qPCR)

Leaves from three stages were selected for RNA extraction using the Total RNA Extraction Kit for Polyphenols and Polysaccharides Plants (Tiangen Biotech, Beijing, China). The RNA concentration was measured using a UV spectrophotometer (BioWave Corporation, Norwalk, CT, USA), and RNA integrity was assessed via 1% TAE agarose gel electrophoresis. The extracted RNA was reverse transcribed into cDNA using a reverse transcription kit (Thermo Fisher Scientific, Waltham, MA, USA). Diluted cDNA was then used as a template for qPCR assay of gene expression levels during leaf development, with *PagPP2AA3* as the internal control. Primers for the 8 *AP2/EREBP* genes were designed for qPCR (Supplementary Table S2). SYBR green was used for qPCR, and the protocol is described in Supplementary Table S3.

## 5. Conclusions

In this study, we found that the *AP2/EREBP* genes in 84K poplar are involved in leaf development and exhibit varying levels of transcriptional expression at different developmental stages. We further analyzed these genes from multiple perspectives, including phylogenetic classification, conserved domains, conserved motifs, gene structure, chromosomal distribution, cis-regulatory elements, and collinearity. These structural characteristics and evolutionary patterns may underlie their functional roles in poplar.

We further screened 76 *AP2/EREBP* genes that exhibited decreasing expression levels as leaves developed to maturity. The expression patterns of eight selected *AP2/EREBP* genes were experimentally validated across three stages of leaf development. Among these, five genes—*Pop_G10G022861, Pop_A01G003858, Pop_A01G081120, Pop_A01G074798,* and *Pop_A07G010900*—showed a decreasing expression trend, suggesting that they may play important roles during early leaf development. The subcellular localization of Pop_A01G003858 and Pop_G11G077730 suggests that they function as regulatory proteins within the nucleus. These findings provide valuable insights for further investigation into the role of this gene family in leaf development.

## Figures and Tables

**Table 1:** Gene IDs from transcriptome sequencing and their corresponding gene IDs in the 84K poplar genome.

Gene IDs from Transcriptome Sequencing	Gene IDs in the 84K Poplar Genome	Sequence Identity
Pop_A08G002307	Pag.A08G002074	99.8
Pop_G10G022861	Pag.G10G000097	100
Pop_A03G013642	Pag.A03G000780	100
Pop_A01G003858	Pag.A01G001847	100
Pop_A01G074798	Pag.A01G001014	100
Pop_A01G081120	Pag.A01G004386	100
Pop_A07G010900	Pag.G07G001107	98.507
Pop_A11G021713	Pag.A11G001270	100
Pop_A06G061616	Pag.A06G002329	91.167
Pop_A04G030612	Pag.A04G001441	100
Pop_G11G077730	Pag.G11G000628	100
Pop_A02G066371	Pag.G02G001276	99.779
Pop_G18G080285	Pag.G18G000982	99.45
Pop_A09G026725	Pag.A09G000992	100
Pop_G08G083366	Pag.G08G000231	100
Pop_G01G075105	Pag.G01G004176	100
Pop_A06G064772	Pag.A06G001287	93.148
Pop_A06G085714	Pag.A06G001642	97.98
Pop_A06G064710	Pag.A06G001129	100
Pop_G10G048209	Pag.G10G001238	100
Pop_G06G051492	Pag.G06G001473	100
Pop_A08G064025	Pag.A08G001341	100
Pop_A14G044652	Pag.A14G000118	99.708
Pop_A04G019568	Pag.A04G000505	100
Pop_G10G048256	Pag.G10G001199	100
Pop_G14G030518	Pag.G14G000066	99.562
Pop_G08G058456	Pag.G08G001259	100
Pop_A06G079386	Pag.A06G001575	100
Pop_A06G079387	Pag.A06G001575	100
Pop_A07G006342	Pag.A07G002351	100
Pop_G06G051489	Pag.G06G001471	100
Pop_A10G069013	Pag.A10G001434	100
Pop_G03G055715	Pag.G03G001984	100
Pop_A10G069049	Pag.A10G001475	100
Pop_G18G078718	Pag.G18G000220	100
Pop_A15G064475	Pag.A15G001243	100
Pop_A06G061913	Pag.A06G002602	98.843
Pop_G15G074700	Pag.G15G001251	100
Pop_A18G013018	Pag.A18G001389	100
Pop_G18G078566	Pag.G18G000401	100
Pop_A16G055344	Pag.A16G000952	100
Pop_A06G079383	Pag.G06G001471	97.403
Pop_G03G021411	Pag.G03G000573	100
Pop_A01G060003	Pag.A01G001290	99.203
Pop_G16G016155	Pag.G16G000943	99.306
Pop_A09G076834	Pag.A09G001392	100
Pop_A03G014855	Pag.A03G000938	100
Pop_A14G044864	Pag.A14G000933	100
Pop_G10G006048	Pag.G10G002382	96.287
Pop_G14G078016	Pag.G14G000872	100
Pop_G03G055651	Pag.G03G002044	100
Pop_A16G055309	Pag.A16G000918	99.309
Pop_A03G019968	Pag.A03G000384	99.15
Pop_A02G012673	Pag.A02G000657	100
Pop_G08G007463	Pag.G08G002278	98.761
Pop_G01G016521	Pag.G01G002018	100
Pop_G03G013809	Pag.G03G001578	100
Pop_G03G010483	Pag.G03G000707	100
Pop_A03G050644	Pag.G03G000707	97.037
Pop_G15G034120	Pag.G15G000130	100
Pop_A12G074013	Pag.A12G001139	100
Pop_G03G013900	Pag.G03G001816	100
Pop_A08G006762	Pag.A08G002372	98.759
Pop_A03G049768	Pag.A03G002081	100
Pop_A01G032318	Pag.A01G001025	100
Pop_G14G044390	Pag.G14G000416	100
Pop_A14G000235	Pag.A14G000466	100
Pop_G12G050880	Pag.G12G000428	100
Pop_A03G050081	Pag.A03G001577	100
Pop_A01G059787	Pag.A01G001128	100
Pop_A01G003802	Pag.A01G001905	100
Pop_A06G063210	Pag.A06G000405	100
Pop_A06G053401	Pag.A06G000772	100
Pop_A02G012335	Pag.A02G001010	86.174
Pop_UnG069776	Pag.A04G001882	72.125
Pop_A06G089378	Pag.G10G001738	56.643

## Data Availability

Data are contained within the article and Supplementary Materials.

## Supplementary Materials

The following supporting information can be downloaded at https://www.mdpi.com/article/10.3390/plants14182842/s1, Table S1: List of primers used for vector construction; Table S2: List of all the primers used in qPCR; Table S3. The protocol of qPCR.
